# Putting the Horse Back in Front: Advancing Simulation Education With Implementation Science and Structured Research Processes

**DOI:** 10.7759/cureus.66799

**Published:** 2024-08-13

**Authors:** Julia Micallef, Krystina M Clarke, Refka Al-Bayati, Samyah Siraj, Adam Dubrowski

**Affiliations:** 1 Health Sciences, Ontario Tech University, Oshawa, CAN; 2 maxSIMhealth Group, Ontario Tech University, Oshawa, CAN

**Keywords:** health professions education, literature review, implementation science methodologies, implementation science, simulation-based education

## Abstract

This editorial explores the application of implementation science methodologies within simulation-based health professions education. It introduces two models, the adapted implementation model for simulation (AIM-SIM) and the implementation quality rubric for simulation (IQR-SIM), tailored to optimize educational simulation programs' development, implementation, and long-term sustainability in simulation contexts. These models are introduced against the backdrop of their development process, which notably lacked a formal needs assessment, highlighting a critical gap in their foundational preparation. To address this gap effectively, the editorial advocates for a scoping review as a strategic next step. The proposed scoping review will aim to comprehensively survey the landscape of existing literature, specifically probing the utilization of implementation science methodologies within simulation-based education. By identifying gaps and assessing the current state of research, the proposed scoping review will seek to substantiate the necessity for a simulation-specific model grounded in implementation science principles. The outcomes of the future scoping review are anticipated to validate the applicability and relevance of AIM-SIM and IQR-SIM in real-world educational settings. Moreover, it may provide insights crucial for refining these models to better meet the dynamic and nuanced needs of the field. By systematically scrutinizing the existing literature, the proposed scoping review may also elude to how effectively current methodologies address the complexities of simulation-based education. Ultimately, this process has the potential to inform future directions in research and practice, ensuring that simulation programs are not only effectively implemented but also sustained over time, thereby maximizing their impact on health professions education.

## Editorial

This editorial explores the application of implementation science [1] methodologies in simulation-based health professions education, addressing the gap between research and practice. It introduces the adapted implementation model for simulation (AIM-SIM) [2] and the implementation quality rubric for simulation (IQR-SIM) [3] as models designed to enhance the implementation and sustainability of simulation programs, highlighting the need for systematic approaches grounded in implementation science principles. Most importantly, however, this editorial also underscores a gap: these two models were developed without a proper need assessment, relying instead on experience and intuition [4]. This editorial proposes a scoping review as a potential remediation strategy [5]. If the scoping review confirms the existence of this gap and demonstrates a need for simulation-tailored, implementation science methodologies to guide simulation program implementation, it will not only justify the use of AIM-SIM and IQR-SIM but also provide directions for enhancing their alignment with the field.

Simulation is crucial in health professions education to set the context, providing learners with realistic, hands-on experiences in a safe and controlled environment [2]. However, implementing successful simulation programs and courses in real-world settings can be challenging due to various factors such as resource constraints, resistance to change, and the complexity of integrating new methodologies into existing curricula [1]. Implementation science, a methodological field dedicated to refining frameworks, models, and methodologies to optimize implementation processes, may play a pivotal role in simulation programming by providing systematic methodologies that facilitate the adoption, integration, and long-term sustainability of educational programs in practice, thereby guiding evidence-based program implementation [1].

This was previously noted by Dubrowski and Dubrowski [2], who highlighted a research-to-practice gap in simulation, noting that translating successful simulation programs from controlled research settings to real-world applications can be challenging without implementing implementation science. One of the key reasons for this underutilization is the lack of awareness and understanding among educators and administrators about how to apply implementation science principles effectively [2]. Using implementation science methodologies, such as frameworks and models, before and during the implementation process can significantly enhance the success of simulation programs by navigating the complexities of real-world settings. These methodologies help identify potential barriers, tailor interventions to specific contexts, and measure implementation outcomes, ensuring that the programs and courses are not only effectively developed or adopted but also sustained over time [2].

As noted earlier, two models were developed to bridge this research-to-practice gap in simulation-based education using implementation science methodologies: AIM-SIM and IQR-SIM. AIM-SIM is a conceptual model structured in three phases: (1) stakeholder engagement and context exploration, (2) preimplementation planning, and (3) program implementation with ongoing evaluation [2]. Each phase involves an implementation team planning and executing well-articulated tasks to plan, build capacity, collect data on the implementation process, and make informed decisions to adjust the course as needed [2]. However, viewing AIM-SIM through a pragmatic lens highlights its shortcomings in translating theory into practical application. While it provides a structured approach across three phases, the model lacks executable methodologies grounded in implementation science. It was noted that a more pragmatic model must complement AIM-SIM by offering actionable steps and tools that facilitate the actual implementation of methodologies derived from implementation science. This complementary model should ensure that the conceptual framework of AIM-SIM is effectively translated into real-world practice, enhancing its utility and impacting the successful implementation of programs and courses in simulation. For this reason, the IQR-SIM was developed as a practical tool to assess the implementation quality of simulation-based courses by identifying gaps, monitoring progress, and supporting the achievement of desired implementation and learning outcomes [3].

However, despite the utility of these conceptual and practical models, it is important to note that these were developed without a formal needs assessment, a step typically considered crucial in model development. Performing a literature review is widely recognized as one of the most effective methods to conduct a needs assessment [4]. By systematically reviewing existing literature, researchers can identify gaps, trends, and best practices within a specific field or topic area. A literature review helps to uncover what has already been studied, what methodologies have been employed, and what knowledge gaps exist. It provides a comprehensive understanding of current practices, challenges, and theoretical frameworks relevant to the subject under investigation. Through this process, researchers gain insights into the needs and priorities within the field, informing the development of targeted interventions, frameworks, or models that address identified gaps and contribute meaningfully to advancing knowledge and practice. Therefore, a literature review serves as a foundational step in conducting a thorough needs assessment, ensuring that subsequent research or initiatives are informed by a robust understanding of the current state of knowledge and practice in the field.

When conducting a literature review, researchers can select from various types based on their research objectives and scope. In the context of simulation and implementation, this editorial examines three common types: systematic reviews, rapid reviews, and scoping reviews, aiming to determine the most suitable approach for addressing this issue. The choice of review approach is critical for obtaining reliable and comprehensive insights.

A systematic review rigorously searches for, evaluates, and synthesizes research evidence according to established guidelines, aiming to provide an impartial summary that minimizes bias through rigorous and transparent procedures [5]. However, this method is resource-intensive and demands a substantial body of literature to ensure reliability. Our initial scan of the literature suggests a lack of such a robust literature base at the intersection of simulation and implementation fields, which may limit the suitability of a systematic review for conducting a needs assessment in this context.

Conversely, a scoping review assesses the breadth and scope of available research literature to outline the nature and extent of evidence, encompassing diverse types, including gray literature [5]. It is proficient at mapping broad topics and identifying gaps in the literature, making it valuable for preliminary assessments to inform further research [5]. However, scoping reviews may prioritize description over evaluation, potentially complicating result interpretation due to variations among included studies. Given the limited and highly diverse literature on implementation science methodologies in simulation, a scoping review may offer a more appropriate approach than a systematic review, allowing for a comprehensive exploration of available evidence and identifying gaps crucial for advancing knowledge and practice in this field.

Rapid reviews expedite the assessment of existing knowledge using streamlined systematic and scoping review methods. However, they focus on efficiency and potentially compromise the methodological rigor and depth of analysis required for complex topics like implementation science in simulation education [5]. The accelerated process may overlook pertinent studies and introduce biases in study selection and data synthesis, limiting their suitability for a comprehensive needs assessment in this context.

Considering the scarcity of systematic evidence on the use of implementation science in simulation-based health professions education, as well as the fact that to the best of our knowledge, it would be the first review of this type conducted, a traditional (i.e., not rapid) scoping review appears most suitable for addressing the question, "what implementation science methodologies, if any, are currently employed in simulation-based health professions education?" Its ability to provide a broad overview and identify gaps in literature will inform the rationale for employing such methodologies and guide improvements and future research directions in this critical area.

Summary

In summary, this editorial discussed the potential of implementation science methodologies to guide the effective implementation of simulation-based education in health professions. It reviewed the development and utility of AIM-SIM and IQR-SIM models, emphasizing the importance of conducting a needs assessment through a literature review, a critical step that has been missed by the researchers to date. That is, the current editorial highlighted a critical gap: both models were developed without a proper needs assessment, relying on experience and intuition. Consequently, this editorial proposed a scoping review as a potential remediation strategy and the next logical step in this research program. If the scoping review confirms the existence of this gap and identifies a need for a simulation-tailored, implementation science methodology to guide simulation program implementation, it will not only justify the use of AIM-SIM and IQR-SIM but also offer insights for improving these models to better align with the needs of the field.

Moreover, another significant contribution of this editorial is to encourage researchers in the field of simulation-based healthcare education to adopt structured research process frameworks, such as the Medical Research Council framework, to develop complex interventions [4]. Such structured approaches are vital for guiding multiyear, multiphased research programs to ensure sequential execution of research phases, thereby maximizing their intended impact. This approach serves as a critical reminder that researchers developing frameworks like AIM-SIM and IQR-SIM should review existing literature and advancements in the field prior to building and applying such models. By doing so, they can ensure that their models are informed by the latest evidence and developments, potentially enhancing their effectiveness in guiding simulation program implementation and sustainability. Therefore, while validating the use of AIM-SIM and IQR-SIM through a scoping review is essential, the broader lesson lies in adopting rigorous research processes to advance the field of simulation-based education effectively.
